# A review of remote ischemic conditioning as a potential strategy for neural repair poststroke

**DOI:** 10.1111/cns.14064

**Published:** 2022-12-22

**Authors:** Wantong Yu, Changhong Ren, Xunming Ji

**Affiliations:** ^1^ Department of Neurology and Beijing Key Laboratory of Hypoxia Translational Medicine Xuanwu Hospital, Capital Medical University Beijing China; ^2^ Center of Stroke, Beijing Institute for Brain Disorder Capital Medical University Beijing China

**Keywords:** ischemic stroke, remote ischemic conditioning, repair

## Abstract

Ischemic stroke is one of the major disabling health‐care problem and multiple different approaches are needed to enhance rehabilitation, in which neural repair is the structural basement. Remote ischemic conditioning (RIC) is a strategy to trigger endogenous protect. RIC has been reported to play neuroprotective role in acute stage of stroke, but the effect of RIC on repair process remaining unclear. Several studies have discovered some overlapped mechanisms RIC and neural repair performs. This review provides a hypothesis that RIC is a potential therapeutic strategy on stroke rehabilitation by evaluating the existing evidence and puts forward some remaining questions to clarify and future researches to be performed in the field.

## Graphical abstract

The figure shows the speculation that chronic RIC may enhance neural repair after ischemic stroke by improving neurogenesis, angiogenesis, axonal regeneration, synaptogenesis and remyelination.

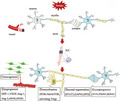

**Abbreviations**
AIS: Acute ischemic stroke
Ang: Angiotensin
BDNF: brain‐derived neurotrophic factor
CNS: central nervous system
eNOS: endothelial nitric oxide synthase
EPO: erythropoietin
GAP‐43: growth‐associated protein 43
HIF‐1: Hypoxia inducible factor‐1
iNOS: inducible nitric oxide synthase
LTD: long‐term depression
LTP: long‐term potentiation
mTOR: mammalian target of rapamycin
Ngb: neuroglobin
NO: nitric oxide
OPC: oligodendrocyte precursor cell
PSD95: Postsynaptic density protein‐95
p‐STAT3: signal transducer and activator of transcription 3 phosphorylation
RIC: Remote ischemic condition
STAT3: signal transducer and activator of transcription 3
SYN: Synaptophysin
Treg: regulatory T cell
VEGF: vascular endothelial growth factor
VEGFR: vascular endothelial growth factor receptor 2

## 1 INTRODUCTION

Stroke is still one of the leading causes of disability and death in the world.^1^ Acute ischemic stroke (AIS) is a disease caused by the occlusion of blood vessel, which results in brain tissue necrosis. Recanalization, including intravenous thrombolysis and endovascular therapy, is the proven treatment for AIS that can reduce mortality. However, there are still a large number of patients having mild to moderate functional deficits at chronic stage after stroke and the burden of stroke is still increasing. This is partly due to the limited treatment option for stroke deficits, which provides incomplete recovery. It is urgent to find effective treatment methods to further improve the functional recovery and reduce the burden of disease. Different from the neuroprotection strategies, which protect brain from ischemia and/or reperfusion injury in the acute stage of stroke (i.e., oxidative stress, excitotoxicity, inflammation),^2^ the targets of neural repair are axonal sprouting, dendritic branching, neurogenesis, axon preservation, remyelination and glial scar reversing.^3^ Therefore, it is critical to develop approaches to augment neural repair to enhance the rate of poststroke functional recovery. Seeking the treatment to combine with rehabilitation will be an effective way. Recent therapeutic strategies under study focus on pharmacotherapy or growth factor that combine with rehabilitation treatment to improve long‐term outcome after stroke.^4^ In addition, physical and non‐invasive strategy are also considered as a promising adjunctive therapy.^5^, ^6^

Remote ischemic condition (RIC) is a non‐invasive procedure by which several episodes of sublethal ischemia and reperfusion in distant organs are applied to trigger endogenous protection of important organs. In the application of RIC, it is common practice to occlude blood flow repetitively of upper limbs in human or hind limbs in rodent bilaterally. The principle of RIC is developed from ischemic conditioning in situ, which was first described by Murry et al. that cycles of myocardial ischemia and reperfusion could protect the heart from a subsequent sustained ischemic injury.^7^ The similar protective effect was subsequently reported in hypoxic conditioning that includes intermittent moderately severe hypoxia or single exposure to acute hypoxia.^8^ The conditioning procedures mentioned above share similar endogenous mechanisms of protection and repair, among which RIC has attracted much attention and emerged as a promising treatment for stroke as it is much safer and more convenient to operate.

RIC has shown an impressive neuroprotective function in ischemic stroke in the past few years. It has been reported that among patients treated with recombinant tissue plasmin, those who received RIC had better prognosis for 90 days than their counterparts who did not.^9^ For patients who failed to receive revascularization therapy within 24 hours after onset, a single episode of RIC provided long‐term protection that significantly reduced 90‐days National Institutes of Health Stroke Scale scores.^10^ Numerous preclinical investigations have also systematically studied the molecular pathways and potential benefits of RIC with promising results. It has been suggested that RIC can significantly reduce the infarct volume, reduce brain edema and improve neurological function.^11^, ^12^, ^13^, ^14^, ^15^ However, the effects of RIC on neural repair after ischemic stroke in the adult brain have yet to be elucidated. In this review, we propose a hypothesis that RIC may be a potential feasible approach for neural repair after ischemic stroke.

## 2 BACKGROUND AND THEORY OF THE HYPOTHESIS

Neural repair after stroke is a dynamic and complex process, wherein a variety of mechanisms are involved. After suffering a vascular event, focal cerebral ischemia will induce neurogenesis, angiogenesis and glial cell activation, thereby creating a favorable environment for endogenous neural repair. However, this endogenous repair in the brain is short‐lasting and light, so it is insufficient to restore neurological function. Therefore, interventions that facilitate neural plasticity by amplifying endogenous mechanisms may accelerate recovery, and this is the principle of most the current complementary treatments in stroke rehabilitation. For instance, physical exercise training, which is considered as an effective therapeutic intervention in stroke recovery, can attenuate neuronal apoptosis and promote neurogenesis,^16^ as well as increase the expression of growth factors such as brain‐derived neurotrophic factor (BDNF),^17^ nerve growth factor,^18^ insulin‐like growth factor‐1 and neurotrophin 4.^19^ Physical training can also alleviate blood–brain barrier dysfunction,^20^ promote angiogenesis and increase cerebral blood flow,^21^, ^22^ increase the density and reuptake ability of astrocytes^23^, ^24^ and promote motor map reorganization,^25^ thus exerting a neural repair effect. Interestingly, RIC has similar underlying mechanisms to physical exercise and may mimic regular exercise.^26^ A number of studies have reported that RIC could amplify neurogenesis and angiogenesis in peri‐infarct area^13^, ^27^, ^28^, ^29^, ^30^ and regulate glial cell function^31^ during stroke recovery. Significantly, early RIC followed by exercise increases mRNA and protein expression of neuroplasticity, synaptogenesis, and angiogenesis related molecules.^32^ In clinical practice, the administration of chronic RIC improved long‐term recovery (modified Rankin Scale score 0–1) and reduced white matter damage in patients with symptomatic atherosclerotic intracranial arterial stenosis (including ischemic stroke and transit ischemic attack).^33^, ^34^ Moreover, RIC could significantly facilitate motor learning in healthy adults, which indicated that RIC might induce neural plasticity.^35^ As of June 13th, 2022, there are two clinical trials at ClinicalTrials.gov investigating the effect of RIC on central nervous system (CNS) rehabilitation are now ongoing (see Table 1). Based on these evidences, we speculate that RIC causes neural repair effect poststroke.

**TABLE 1:** Representative Clinical Trials of Remote Ischemic Conditioning for rehabilitation in ClinicalTrials.gov

Study	N	Participants	RIC protocol	Primary outcome	Status	ClinicalTrials.gov Identifier
Remote Ischemic Conditioning, Bimanual Skill Learning, and Corticospinal Excitability	30	Children with unilateral cerebral palsy	5 × 5 min inflation/deflation of cuff Cuff pressure: 20 mmHg above systolic blood pressure to 250 mmHg	Change in Assisting Hand Assessment, Bimanual Task Performance, Resting and Active Motor Thresholds and Stimulus–response curves	Recruiting	NCT05355883
Remote Ischemic Conditioning for Motor Recovery After Acute Ischemic Stroke	20	Patients with AIS	5 × 5 min inflation/deflation of cuff Cuff pressure: 200 mmHg	Changes in Fugl‐Meyer score	Recruiting	NCT05263531

Abbreviations: AIS, acute ischemic conditioning; RIC, remote ischemic conditioning.

## 3 EVALUATION OF THE HYPOTHESIS

There are numerous mechanisms involved in neural repair to improve recovery poststroke, and some of them also exist in the potential mechanisms of RIC. Here, these overlapping ones are presented to support the hypothesis, in which both direct evidence and indirect evidence are contained.

### 3.1 Neurogenesis

Neurogenesis after stroke is a process by which neural progenitor cells generate and differentiate into mature neurons, migrate to infarct area and integrate to existing neuronal circuits,^36^ which significantly contributes to the brain function recovery poststroke. It is unclear whether RIC promote neurogenesis after stroke. Esposito et al. reported that ischemic postconditioning in situ significantly increased the number of newborn neurons and improved neurological outcomes.^27^ Furthermore, trophic factors enhance neurogenesis in the stroke brain.^37^ hypoxic conditioning could promote the proliferation and migration of endogenous neuronal precursor in rodents by increasing the expression of BDNF^38^ and regulating metabolism of neural stem cells (decreasing fatty acid oxidation and increasing glycolysis).^39^ Hypoxia inducible factor‐1 (HIF‐1) is well known to stimulate neurogenesis and neuronal differentiation, and one important pathway contributing to the neurogenesis effect of HIF‐1 involves induction of neuroglobin (Ngb).^40^ Ngb has a function of elevating oxygen distribution to the neurons. It promotes both proliferation and neuronal differentiation of neural progenitor cells by enhancement of Wnt signaling, thereby promoting neurogenesis after stroke.^41^ Ren et al. found that a single episode of RIC at onset of cerebral ischemia (per‐conditioning) combined with daily RIC initiated after reperfusion (postconditioning) during the 14 days could significantly increase Ngb expression in the peri‐infarct region.^13^ Therefore, we assume that RIC could amplify and provide long‐term induction of neurogenesis.

### 3.2 Angiogenesis

It has been reported that blood vessels could create a microenvironment, which is suitable for neurogenesis by supplying oxygen, nutrients, and soluble factors.^42^ It acts as scaffold for neural progenitor cells to migrate to damaged region.^43^ Functional recovery after stroke is accompanied by revascularization in perilesional areas.^44^ Therefore, treatments that promote angiogenesis play a critical role in the neural repair process. RIC has been found to promote angiogenesis after stroke. Ren et al. demonstrated that remote ischemic per‐conditioning followed by postconditioning for 7 or 14 days could promote arteriogenesis and increase cerebral blood flow and collateral circulation after ischemic stroke in the rat through increasing vascular Notch signaling activity.^29^ In addition, they reported that RIC significantly increased the expression of endothelial nitric oxide synthase (eNOS) and regulated angiogenesis by eNOS/nitric oxide (NO) pathway after chronic cerebral hypoperfusion.^30^ Consistently, Khan et al. found that long‐term daily RIC therapy (1 or 4 months) significantly enhanced both angiogenesis and arteriogenesis in mouse bilateral carotid artery stenosis model.^45^, ^46^ Moreover, RIC could enhance the expression of angiogenesis related factors. For instance, vascular endothelial growth factor (VEGF) is the key factor to proangiogenic activity. It could increase the vascular permeability, promote endothelial cell migration and survival when binding to VEGF receptor 2 (VEGFR2).^47^ For in vivo experiment, the expression of HIF‐1α and VEGF was significantly increased by RIC at the mRNA and protein levels.^32^, ^48^, ^49^, ^50^ Additionally, exosomes derived from RIC could increase the expression of eNOS, inducible nitric oxide synthase (iNOS), HIF‐1α, Angiotensin 1 (Ang‐1), and VEGF in rats.^51^ Ischemic postconditioning in situ also upregulate the VEGF within peri‐infarct regions.^52^ Hence, RIC facilitates recovery possibly by enhancing angiogenesis.

### 3.3 Axonal regeneration

Neurogenesis and angiogenesis are the structural basis of neural repair, while functional connections between newborn and surviving neurons need to be established to realize effective neurological function recovery. The action potentials between neurons are conducted by axons, therefore, axonal regeneration and reconnection play a central role in poststroke recovery. In the central nervous system, the influence of RIC on axonal regeneration has not been tested yet, but encouraging results confirmed that hypoxic conditioning significantly increased the numbers of thinly myelinated regenerating axons in surgically repaired peripheral nerves.^53^ Axon sprouting can be enhanced or limited by several transcription factors, cytokines, chemokines, growth factors, and other molecules. Signal transducer and activator of transcription 3 (STAT3) is a transcription factor, which promotes the regrowth of damaged axons in adult central nervous system.^54^ Many cytokines and neurotrophic factors play a role in promoting neurite extension through activation of STAT3.^55^ Hildebrandt et al. recruited healthy volunteers to receive RIC and collected their venous blood to obtain dialysates before and after conditioning. The analysis result suggested that RIC significantly increased the level of STAT3 phosphorylation (p‐STAT3) compared to baseline.^56^ Youn et al. also reported that p‐STAT3 were increased by RIC,^57^ which indicated that RIC may activate STAT3. Growth‐associated protein 43 (GAP‐43) is also an important marker related to the structural integrity of neurons, which is known to promote axonal regeneration by guiding growth cones,^58^, ^59^, ^60^ It was reported that mRNA and protein expressions of GAP‐43 could be increased by RIC in rats after cerebral ischemia.^61^, ^62^ Similarly, intermittent hypoxia (6 min FiO_2_ = 6% with 6 min normoxia, 8 h per day) applied for 4 weeks increased the expression of GAP‐43 in young rats.^63^ Additionally, prior studies suggest that erythropoietin (EPO) induces axonal sprouting via activating cellular JAK2 and PI3K signaling.^64^ Hypoxic conditioning has been discovered to increase EPO levels in healthy individuals serum^65^ and rat brains.^66^ Mammalian target of rapamycin (mTOR) is a protein kinase, which regulate cell growth, survival and metabolism. In the CNS, mTOR also act as a nutrient sensor that support neuronal growth and plasticity.^67^ mTOR pathway is a crucial signaling pathway, which can promote axon regeneration,^68^ as the activation of mTOR may promote the synthesis of the raw materials for axon extension.^69^ However, RIC has been proved to downregulate mTOR activity and upregulate miRNAs, which repress mTOR expression and signaling.^70^, ^71^ In this regard, the effect of RIC on axon regeneration need to be further explored.

### 3.4 Synaptogenesis

Neuronal synapse formation and maturation are complex and regulated through multiple steps. In recent years, RIC has been discovered to improve synaptogenesis in stroke model. Wang et al. found that 3 cycles of RIC after cerebral artery occlusion could improve synaptogenesis in ischemic penumbra.^61^ Moreover, there are several markers that monitor synaptogenesis. It was reported that serine 41 on GAP43 could regulate synaptic plasticity when it was phosphorylated.^72^ Postsynaptic density protein‐95 (PSD95) is a major synaptic scaffolding molecule, which controls synaptic transmission^73^ and can be used to label postsynaptic terminals. Synaptophysin(SYN), a protein located in neurotransmitter vesicles, can identify presynaptic terminals. Geng et al. reported that RIC significantly increased mRNA and protein expressions of GAP43, SYN and PSD‐95 compared to stroke‐only rats, indicating a potential role in promoting synaptogenesis.^62^ Dendritic spines can respond to many events with rapid changes as long‐term potentiation (LTP) and long‐term depression (LTD). Neurotrophic factors play critical roles in LTP thus enhancing synaptic plasticity. Among all neurotrophins, BDNF distribute most widely in mammalian brain and modulate broad range of process including synaptic plasticity and remodeling.^74^ RIC has shown to upregulate BDNF significantly after ischemic reperfusion injury.^75^ In addition to RIC, similar protective effects of hypoxic conditioning were also described in synaptic connection. Tsai et al. demonstrated that hypoxic conditioning applied after cerebral ischemia increased hippocampal functional synaptogenesis via BDNF expression.^76^ The studies mentioned above may support the hypothesis that RIC is an adjunct treatment in synaptic alteration.

### 3.5 Remyelination

Damaged oligodendrocytes in the acute phase cause myelin loss, which is the leading cause of white matter dysfunction. Remyelination is one of the key processes in neural repair after ischemic stroke,^77^ which is mediated by oligodendrocyte precursor cells (OPC) proliferation and differentiation at sites of infarct with demyelination.^78^ Oligodendrogenesis can occur in peri‐infarct areas in repair processes companied with angiogenesis and neurogenesis. So far, no studies have investigated the influence of RIC on poststroke remyelination. Nevertheless, OPCs communicated closely with endothelial cells in the oligovascular niche, wherein endothelial cells support the survival and proliferation of OPC.^79^, ^80^ RIC is proven to promote angiogenesis poststroke, therefore, RIC may be beneficial to remyelination. Notably, in chronic cerebral hypoperfusion rats, Li et al. showed that RIC for 28 days promoted myelination by inhibiting oligodendrocytes apoptosis in the corpus callosum.^81^ Daily RIC for 2 weeks in mice also ameliorated the loss of myelin basic protein caused by chronic cerebral hypoperfusion.^46^ The above results suggest that RIC has a potential positive influence on poststroke remyelination. Furthermore, infiltration of peripheral immune cells into ischemic area also contribute to poststroke repair.^82^ Over the last decade, several studies have described the infiltration of T cells in CNS after stroke,^83^, ^84^ demonstrating that regulatory T cells (Treg) promote oligodendrocyte differentiation and remyelination.^85^ Shi et al. reported that the function of Treg cells in facilitating oligodendrogenesis and remyelination was mediated by promoting tissue‐reparative microglia responses following crosstalk with microglia at the chronic stage of stroke.^86^ It was reported that ischemic postconditioning (reperfusion for 10 min after which the middle cerebral artery was reoccluded for 10 min) for one episode can facilitate to shift microglia into tissue repairing forms,^52^ which actively participate in myelin repair.^87^, ^88^ In addition, a recent study confirmed the contribution of RIC is linked to Treg cells thriving. The increased quantity of Treg cells by 3 cycles of RIC immediately after middle cerebral artery reperfusion was showed in mice and directly linked to improvement of neurological function.^89^ Hence, we suggest that RIC can enhance poststroke remyelination through directly acting on oligodendrocytes and modulating microglial response.

## 4 CHALLENGES AND FUTURE DIRECTIONS FOR THE STUDY OF RIC IN POSTSTROKE REHABILITATION

After summarizing the evidence that RIC can enhance neural repair (see Table 2), the positive effects of RIC on stroke rehabilitation were put forward, but the hypothesis is needed to be test in stroke models. To clarify whether RIC facilities the process of neural repair, there are still some questions and perspectives need to be noted in further studies.

**TABLE 2:** The possibly overlapping mechanism between RIC and neural repair in preclinical studies.

Study	Type of animal	Model	Protocol of RIC or other form of conditioning	Possible mechanism	Direct/indirect evidence
Neurogenesis					
Esposito^27^	Male Sprague–Dawley rats	MCAO	100‐minute middle cerebral artery occlusion plus 10‐minute reperfusion plus 10‐minute reocclusion	NR	indirect
Zhu^38^	Male Sprague–Dawley rats	‐	Expose to pressure equivalent to an altitude of 5000 m for 4 h, then reach the normal level in 15 min, once daily for 14 days	BDNF–TrkB signaling	indirect
Li^39^	Male C57BL/6 mice	MCAO	After reperfusion, 8% O_2_ for 3 h, once daily until sacrifice	decreased FAO and increased glycolysis	indirect
Ren^13^	Male Sprague Dawley rats	MCAO	3 × 10 min occlusion or release, immediately following ischemia and once daily for 14 days	promotes neuroglobin expression	indirect
Angiogenesis					
Ren^30^	Male Sprague–Dawley rats	2VO	3 × 10 min occlusion or release, once daily for 28 days	eNOS/NO pathway	direct
Ren^29^	Male Sprague–Dawley rats	MCAO	3 × 10 min occlusion or release, immediately following ischemia and once daily for 7 or 14 days	Notch 1 signaling	direct
Khan^45^	Male C57BL/6 mice	BCAS	4 × 5 min occlusion or release, once daily for 1 or 4 months	NR	direct
Khan^46^	Male C57BL/6 mice	BCAS	4 × 5 min occlusion or release, once daily for 2 weeks	NR	direct
Wang^32^	Male Sprague–Dawley rats	MCAO	6 h after reperfusion, 3 × 10 min occlusion or release, once daily for 28 days	promotes VEGF, Ang‐1, Ang‐2 expression	direct
Chen^51^	Male Sprague–Dawley rats	myocardial infarction	NR	Increases eNOS, iNOS, HIF‐1α, Ang‐1, and VEGF expression by exosomes	indirect
Esposito^52^	Male Sprague–Dawley rats	MCAO	100‐minute middle cerebral artery occlusion plus 10‐minute reperfusion plus 10‐minute reocclusion	promotes VEGF expression in peri‐infarct area	direct
Axonal regeneration					
Huang^94^	Male Sprague–Dawley rats	Hemorrhagic shock	4 × 5 min occlusion or release,	increases the phosphorylation levels of STAT3	indirect
Wang^61^	Male Sprague–Dawley rats	MCAO	3 × 10 min occlusion or release, once after reperfusion	Promotes GAP43 expression	direct
Geng^62^	Male Sprague–Dawley rats	MCAO	3 × 10 min occlusion or release	Promotes GAP43 expression	direct
Chen^63^	Male Sprague–Dawley rats	dMCAO	3 cycles of 30 s reperfusions and 10 s occlusions of the bilateral CCAs, once after reperfusion	Promotes GAP43 expression	indirect
Coimbra‐Costa^66^	Male Sprague–Dawley rats		Exposure to pressure equivalent to an altitude of 4000 m for 4 h, once daily for 8 days	increase EPO levels in the brain	indirect
Synaptogenesis					
Wang^61^	Male Sprague–Dawley rats	MCAO	3 × 10 min occlusion or release, once after reperfusion	Promotes SYN, PSD95 expression	direct
Geng^62^	Male Sprague–Dawley rats	MCAO	3 × 10 min occlusion or release	Promotes SYN, PSD95 expression	direct
Tsai^76^	Male Sprague–Dawley rats	MCAO	12% O_2_ for 4 hours, once daily for 7 days	promotes BDNF expression	indirect
Remyelination					
Li^81^	Male Sprague–Dawley rats	2VO	3 × 10 min occlusion or release, once daily for 28 days	PI3K/Akt/mTOR pathway	direct
Khan^45^	Male C57BL/6 mice	BCAS	4 × 5 min occlusion or release, once daily for 2 weeks	NR	direct
Yu^89^	Male C57BL/6 mice	MCAO	3 × 10 min occlusion or release, once after reperfusion	activating and maintaining the Tregs	indirect

*Note*: Direct evidence: the intervention is RIC instead of ischemic conditioning in situ or hypoxic conditioning, and the result can indicate the effect of RIC on neural repair processes directly instead of related processes. Indirect evidence: studies that do not meet the definition of direct evidence.

Abbreviations: 2VO, 2‐vessel occlusion; Ang, angiotensin; BCAS, bilateral carotid artery stenosis; BDNF, brain‐derived neurotrophic factor; eNOS/NO, endothelial nitric oxide synthase /nitric oxide; EPO, erythropoietin; FAO, fatty acid oxidation; GAP43, growth‐associated protein 43; MCAO, middle cerebral artery occlusion; NR, not reported; PSD95, postsynaptic density 95; RIC, remote ischemic conditioning; STAT3, signal transducer and activator of transcription 3; YN, synaptophysin; Treg, regulatory T cells; TrkB, tropomyosin related kinase B; VEGF, vascular endothelial growth factor.

Firstly, it is important to identify suitable time window for RIC in stroke rehabilitation, such as when to begin treatment. It is worth noting that four‐week chronic RIC initiated on the fifth day of cerebral ischemia has failed to improve the neurobehavioral deficit in rats endothelin‐1 stroke model.^90^ Thus RIC initiated at chronic stage seems to have no advantage in function improvement. Meanwhile, acute stage application of RIC is proved to be effective, so it may be beneficial to apply RIC as early as possible and provide treatment until the chronic phase of stroke in further animal studies.

Secondly, how long RIC treatment should last in stroke rehabilitation needed to be considered as well. As a non‐invasive and effective conditioning strategy, RIC has been widely investigated clinically in the acute phase of ischemic stroke. The results of these studies emphasized the neuroprotection role of short‐term RIC at the beginning of stroke onset, but the effect of chronic RIC application on stroke patients remain unclear. Poalelungi et al. evaluated the effect of RIC applied in first 5 days after onset of patients, and results suggested that RIC partly improved disability and cognition without statistical difference.^91^ Similarly, there are no significant improvements in infarct size or clinical outcome in patients who receive RIC for 4 days after stroke.^92^ Conversely, 1‐year application of RIC significantly improved cerebral blood flow and slowed the arterial progression of the stenotic‐occlusive lesions in patient with Moyamoya disease.^93^ These findings suggest that for the long‐lasting process like vascular remodeling in Moyamoya disease and neural repair, chronic daily RIC may be a more appropriate strategy in further clinical studies. Additionally, besides the assessment of function, neuroimaging is needed during recovery to evaluate whether distributed structural and functional connectivity can be changed by RIC.

## 5 CONCLUSION

More and more evidence has emerged to identify the influence of RIC on stroke recovery. Although there is still a lack of direct evidence to support the repair effects of RIC on ischemic stroke, these preclinical results have given the insight that the potential positive effects of RIC on neural repair, including neurogenesis, angiogenesis, axon regeneration, synaptogenesis and remyelination. More preclinical and clinical research work is needed to support the use of RIC as a promising therapy in stroke rehabilitation.

## CONFLICT OF INTEREST

The author(s) declared no potential conflicts of interest with respect to the research, authorship, and/or publication of this article.

## Data Availability

Data sharing not applicable to this article as no datasets were generated or analyzed during the current study.
